# Prognostic Utility of the Ki-67 Labeling Index in Follicular Thyroid Tumors: a 20-Year Experience from a Tertiary Thyroid Center

**DOI:** 10.1007/s12022-022-09714-4

**Published:** 2022-03-19

**Authors:** L. Samuel Hellgren, Adam Stenman, Johan O. Paulsson, Anders Höög, Catharina Larsson, Jan Zedenius, C. Christofer Juhlin

**Affiliations:** 1 Department of Oncology-Pathology, Karolinska Institutet, Stockholm, Sweden; 2 Department of Pathology and Cancer Diagnostics, Karolinska University Hospital, Stockholm, Sweden; 3 Department of Breast, Endocrine Tumors and Sarcoma, Karolinska University Hospital, Stockholm, Sweden; 4 Department of Molecular Medicine and Surgery, Karolinska Institutet, Stockholm, Sweden

**Keywords:** Follicular thyroid adenoma, Follicular thyroid carcinoma, Ki-67, Immunohistochemistry, Prognosis

## Abstract

Follicular thyroid tumors pose a diagnostic challenge on the preoperative level, as the discrimination between follicular thyroid carcinoma (FTC) and adenoma (FTA) demands careful histopathological investigation. Moreover, prognostication of FTCs is mostly based on tumor size and extent of invasive properties, while immunohistochemical markers pinpointing high-risk cases are lacking. We have routinely established a Ki-67 labeling index for follicular thyroid tumors since 1999. To assess the potential value of Ki-67 as an adjunct tool to (1) correctly separate FTCs from FTAs and (2) help identify poor-prognosis FTCs, we collected histopathological and clinical data from 818 follicular thyroid tumors with a histological Ki-67 labeling index established in clinical routine practice (516 FTAs, 252 FTCs, and 50 follicular thyroid tumors of uncertain malignant potential (FT-UMPs)). The Ki-67 labeling index was higher in FTCs (mean 5.8%) than in FTAs (mean 2.6%) (*P* < 0.001), and a receiver operating characteristic curve analysis revealed a cut-off value of 4% to separate FTC from FTA with a sensitivity and specificity of 65% and 83%, respectively. Similarly, a Ki-67 labeling index above 4% was found to identify FTCs that later metastasized from clinically indolent FTCs with a sensitivity and specificity of 80% and 48%, respectively. Ki-67 constituted an independent predictor of future FTC metastases/recurrence and death of disease, and a value > 4% was a reliable prognostic marker within individual pT staging groups. We conclude that Ki-67 is a potentially valuable marker for the prognostication of FTCs, and future implementation in the histopathological assessments of follicular thyroid tumors could be beneficial if reproduced in international series.

## Introduction

From a diagnostic and prognostic perspective, there is no doubt that follicular thyroid tumors constitute one of the most clinically challenging entities in terms of thyroid neoplasia. As the preoperative assessment is to date unable to reliably differentiate between follicular thyroid carcinoma (FTC) and adenoma (FTA), this distinction is reserved for the surgical pathologist when assessing invasive properties using standardized histopathology [1]. However, even in the hands of an experienced diagnostician, these lesions are sometimes notoriously difficult to evaluate, not least exemplified by the borderline category “follicular tumors of uncertain malignant potential” (FT-UMPs) [1]. In addition to the present diagnostic limitations, the prognostication of an FTC patient is based on the age at surgery, the overall TNM stage, and the histological subtyping of tumors into either minimally invasive (miFTC), encapsulated angioinvasive (eaiFTC) or widely invasive (wiFTCs) [1, 2]. Even so, the histological subtyping is not always straightforward and might potentially differ between institutions, and immunohistochemical markers indicative of invasive behavior are generally lacking in the clinical setting, although several proteins have been previously proposed [3–7].

In endocrine pathology, the Ki-67 proliferation labeling index is a well-known marker, not least exemplified by the ability to grade gastro-entero-pancreatic neuroendocrine tumors into subgroups of prognostic and therapeutic relevance [1]. Moreover, Ki-67 has also been proven valuable for highlighting the metastatic potential in pheochromocytomas and abdominal paragangliomas, adrenocortical tumors, and parathyroid tumors [8–13]. Although there is currently no established role for Ki-67 in the diagnostic or prognostic work-up of thyroid tumors according to the 2017 World Health Organization (WHO) criteria, a higher proliferation index has been reported in cases with poor outcome [1, 14–23]. Ki-67 could therefore be considered a potentially helpful marker when assessing thyroid neoplasia from a prognostic perspective. In terms of follicular thyroid tumors, a Ki-67 labeling index of > 5% has been reported to predict worse outcome in minimally invasive FTCs, and a preoperative Ki-67 labeling index > 5% in fine needle aspiration biopsies (FNABs) has been associated with FTCs as opposed to FTAs [15, 24]. At our department, most follicular thyroid tumors have been assessed by Ki-67 immunohistochemistry since the methodology was established in clinical routine practice in 1999, and the labeling index has been an integrated part of the pathology report whenever an FTC is signed out. We therefore sought to investigate the potential of this marker in the diagnostic and prognostic context by collecting data from a large cohort of follicular thyroid tumors with long-term follow-up data.

## Methods

### Study Cohort

Since this is a purely retrospective analysis, we included all follicular thyroid tumors (solitary or multifocal) surgically removed and diagnosed at Karolinska University Hospital, Solna, Sweden, for which a Ki-67 labeling index was listed in the final pathology report. Second-opinion cases from outside hospitals were excluded to avoid referral bias. The tumor cohort (*n* = 818) included 516 FTAs, 50 FT-UMPs, and 252 FTCs, starting in 1999 when the Ki-67 immunostaining was first clinically available at our department. During 1999–2014, a Ki-67 labeling index was acquired for all diagnosed FTCs as well as subsets of FTAs at the discretion of the responsible endocrine pathologist at that time (most often larger tumors). From 2015 and onwards, the procedure was standardized to encompass all follicular thyroid tumors, irrespective of tumor type or size. All tumors entitled “atypical follicular thyroid tumors” before the advent of the FT-UMP terminology in the 2017 WHO classification were re-investigated according to these FT-UMP criteria by two pathologists (LSH and CCJ) [1].

### Reviews of Pathology Reports and Medical Charts

Our department is a tertiary center for thyroid surgery with a catchment area of approximately 2.3 million inhabitants and > 1000 thyroidectomies performed annually. We screened our institutional electronic pathology database for follicular thyroid tumors diagnosed at the Karolinska University Hospital in Solna with an electronic search function using Systematized Nomenclature of Medicine (SNOMED) codes T96 (for thyroid, unspecified lobe), M833 and M823 (for follicular thyroid tumors), M829 (for oncocytic tumors), and M814 (for “adenomas”). All pathology reports were read by a single author (LSH) and histopathological variables for all cases with an established Ki-67 labeling index were retrieved, including diagnosis, tumor size, type of invasion (capsular/vascular), extrathyroidal extension (ETE), and the pTNM cancer stage [2]. Angioinvasion was defined as an intravascular tumor thrombus with associated fibrin within the outer layers or beyond the contour of the tumoral capsule. If findings were equivocal, level sectioning, van Gieson stains, and/or endothelial cell immunohistochemical markers were usually procured. Only non-functioning, *bona fide* follicular tumors were included in the study, thereby excluding toxic adenomas, hyalinizing trabecular tumors, tumors with a poorly differentiated component, and/or co-existing papillary or medullary thyroid cancer. Well-differentiated thyroid tumors of uncertain malignant potential (WDT-UMPs) were also excluded from the study as the true origin of these lesions is debated. All FTCs were re-classified to adhere to the most recent WHO terminology with three subgroups (miFTC, eaiFTC, wiFTC) [1]. Furthermore, since oncocytic tumors were introduced as a separate entity in 2017, pathology reports prior to this year contain a mix of conventional follicular thyroid tumors as well as “oxyphilic” or “Hürthle cell” follicular thyroid tumors. We tackled this issue by assigning a status of “oncocytic differentiation” to all tumors exhibiting these features irrespective of which WHO classification they were originally diagnosed under. Another author (AS) reviewed the medical charts for all included patients and listed conventional clinical parameters, including age at surgery, sex, metastatic disease, and patient outcomes. Tumor recurrences and distant metastases were documented through radiology for all cases, as well as serum thyroglobulin measurements for subsets of cases.

### Tissue Handling and the Ki-67 Labeling Index

The advantage of a retrospective mono-institutional analysis using clinical routine Ki-67 reporting is the consistency of tissue handling. At our department, thyroid tumors are macroscopically handled by subspecialized endocrine pathologists, and the vast majority of tumors included in this study has been cut and sampled by two individuals, which are both authors of this paper. The tissue handling protocol follows a standardized format, in which samples are macroscopically investigated directly after excision, then fixated in formalin. After proper fixation, specimens are cut with a 5-mm thickness and sampled for histological investigations.

The Ki-67 immunohistochemistry and scoring procedures for postoperative specimens at our department have followed a consistent model since its launch in 1999. From each case, a representative section of the primary tumor was selected for subsequent Ki-67 immunohistochemistry using a standardized methodology in a clinical laboratory setting at our department. Sections of lymph nodes were used as positive controls, and various de-identified parenchymatous tissues were used as negative controls. Between 1999 and 2002, the immunohistochemical staining was performed through manual routine by a small group of experienced lab technicians using the avidin–biotin complex Vectastain method (Vector Laboratories, Burlingame, CA, USA). From 2003 onwards, automated staining procedures were used; first the BenchMark XT followed by the BenchMark ULTRA (Ventana Medical Systems, AZ, USA) in 2009. From 1999 to 2015, the Mib-1 antibody clone (Immunotech, Marseille, France) was used as a primary antibody, but was replaced in 2016 by the CONFIRM anti-Ki-67 antibody (clone 30–9, Roche, Basel, Switzerland). Since no official guidelines for establishing a Ki-67 labeling index in thyroid tumors exist, the scoring methodology was established in 1999 as manual counting of 2000 cells using hot spot areas and an ocular grid, based on contemporary guidelines used for neuroendocrine tumors [25]. This procedure was consistently applied throughout the study period irrespective of the staining methodology or antibody clone used. Representative sections were chosen at random, but in FTCs often coincided with the area of invasion (data not shown). Hot spot areas were chosen through visual inspection at low power magnification. The vast majority of cases from the entire study period was signed out by two dedicated endocrine pathologists (AH and CCJ). To confirm the reproducibility of the method, subsets of cases from different time periods were retrieved and re-counted by a single pathologist (CCJ), and the blinded results were then compared to the original pathology reports.

### Comparisons to *Telomerase Reverse Transcriptase* (*TERT*) Promoter Mutations and Gene Expression

In terms of molecular risk stratification of follicular thyroid tumors, *TERT* gene aberrancies are recognized as markers of worse clinical outcomes [26–28]. We included molecular data from 62 FTCs regarding *TERT* promoter mutations using Sanger sequencing and *TERT* gene expression from quantitative real-time PCR analyses derived from an earlier publication, and correlated these parameters to the Ki-67 indices [29].

### Statistical Analyses

The statistical analyses were performed using IBM SPSS Statistics version 27 (SPSS Inc, Chicago, IL, USA). Chi-square test was used to compare differences in categorical variables such as the presence of metastasis/recurrent disease, death of disease, and associations to *TERT* promoter mutations and *TERT* gene expression. Mann–Whitney *U*-test was used to compare differences between continuous variables such as size, age at surgery, and Ki-67 labeling index. The Kruskal–Wallis test was used to compare differences between multiple subgroups of FTC. Cox regressions were used in both univariate and multivariate analyses in order to find predictors of metastasis/recurrence and death of disease. A receiver operating characteristic (ROC) curve and area under the curve (AUC) analyses were used to establish cut-offs for Ki-67 labeling indices and to calculate sensitivity and specificity for these cut-off values. Survival analyses were performed with a log-rank test and illustrated with Kaplan–Meier plots for the endpoints time to metastatic event/recurrent disease (disease-free survival) and time to death of disease. Cohen’s kappa test was applied to test for reproducibility of the Ki-67 scoring procedure. For all analyses, *P* values < 0.05 were considered statistically significant.

## Results

### General Characteristics of the Study Cohort

**Fig. 1:**
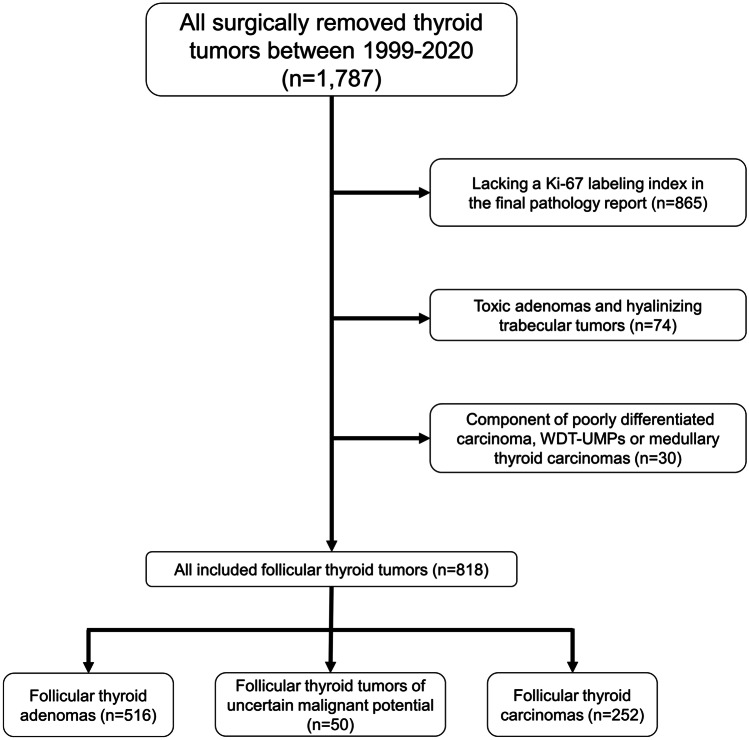
Study inclusion flow chart. General overview of the selection process regarding follicular thyroid tumors with an established Ki-67 labeling index. WDT-UMPs, well-differentiated thyroid tumors of uncertain malignant potential, *n*, sample size

The procedure for tumor inclusion is illustrated in Fig. 1. A total of 1787 thyroid tumors were retrieved using SNOMED-based searches in our institutional pathology database. We excluded 865 tumors due to the lack of a Ki-67 index (841 adenomas and 24 carcinomas), 74 tumors since they were toxic adenomas or hyalinizing trabecular tumors, and 30 tumors since they displayed either a component of poorly differentiated carcinoma, WDT-UMP, or medullary thyroid carcinoma. In total, 818 follicular tumors derived from 809 patients with a Ki-67 labeling index listed in the pathology report were included in the study (516 FTAs, 50 FT-UMPs and 252 FTCs) (Fig. 1). The histological and clinical parameters for the 818 tumors in the study are summarized in Table 1. FTCs were subclassified as either miFTC (*n* = 120), eaiFTC (*n* = 34), wiFTC (*n* = 95), or FTCs with a predominant macrofollicular growth pattern (*n* = 2). One tumor lacked sufficient information in order to be subclassified into a certain growth pattern subgroup. Seventy-five tumors displayed oncocytic differentiation. The mean follow-up time for all FTCs was 68.5 months, ranging from 2 to 255 (median 54) months. A total of 47 patients exhibited distant metastases or local recurrences at follow-up (45 FTC patients and 2 FT-UMP patients), and 20 died of disease (19 FTC patients, 1 FT-UMP patient). The FTCs were in general larger than FTAs (41 *vs* 31 mm, *P* < 0.001), and patients with FTC were generally older than those with FTA (55 *vs* 50 years, *P* < 0.001). FTCs and FT-UMPs did not differ significantly regarding size (41 *vs* 39 mm, *P* = 0.87), or regarding age at surgery (55 *vs* 53 years, *P* = 0.28). The FT-UMPs were, like FTCs, in general larger than FTAs (39 *vs* 31 mm, *P* < 0.001) but did not differ significantly regarding age at surgery (53 *vs* 50 years, *P* = 0.21).

**Table 1:** Histological and clinical parameters of all included tumors

	**FTC**	**FT-UMP**	**FTA**
Number of tumors, n	252	50	516
Mean age at surgery, years (range)	55.2 (10–92)	52.6 (20–86)	49.7 (13–93)
Female:male	178:74	41:9	405:111
Mean tumor size, mm (range)	41.3 (9–120)	39.3 (7–75)	30.5 (3–100)
Oncocytic differentiation, *n*	75 (29.8%)	12 (24%)	143 (27.7%)
Mean Ki-67, % (range)	5.8 (1–32)	5.1 (1–14)	2.6 (0.5–17)
pT1, *n*	33 (13.2%)		
pT2, *n*	104 (41.6%)		
pT3, *n*	110 (44%)		
pT4, *n*	3 (1.2%)		
Extrathyroidal extension, *n*	10 (4.1%)		
Vascular invasion, *n*	125 (50.4%)		
Capsular invasion, *n*	221 (87.7%)		
Mean follow-up time, months (range)	68.5 (2–255)		
Death of any cause, *n*	41 (16.3%)	2 (4%)	28 (5.4%)
Death of disease, *n*	19 (7.5%)	1 (2%)	0 (0%)
Metastasis/recurrent disease, *n*	45 (17.9%)	2 (4%)	0 (0%)

*FTA* follicular thyroid adenoma, *FT-UMP* follicular tumor of uncertain malignant potential, *FTC* follicular thyroid carcinoma, *pT1-4* pathological tumor stage according to the American Joint Committee on Cancer (AJCC) Cancer Staging Manual 8^th^ edition, *n* sample size

### Diagnostic Properties of the Ki-67 Labeling Index

**Fig. 2:**
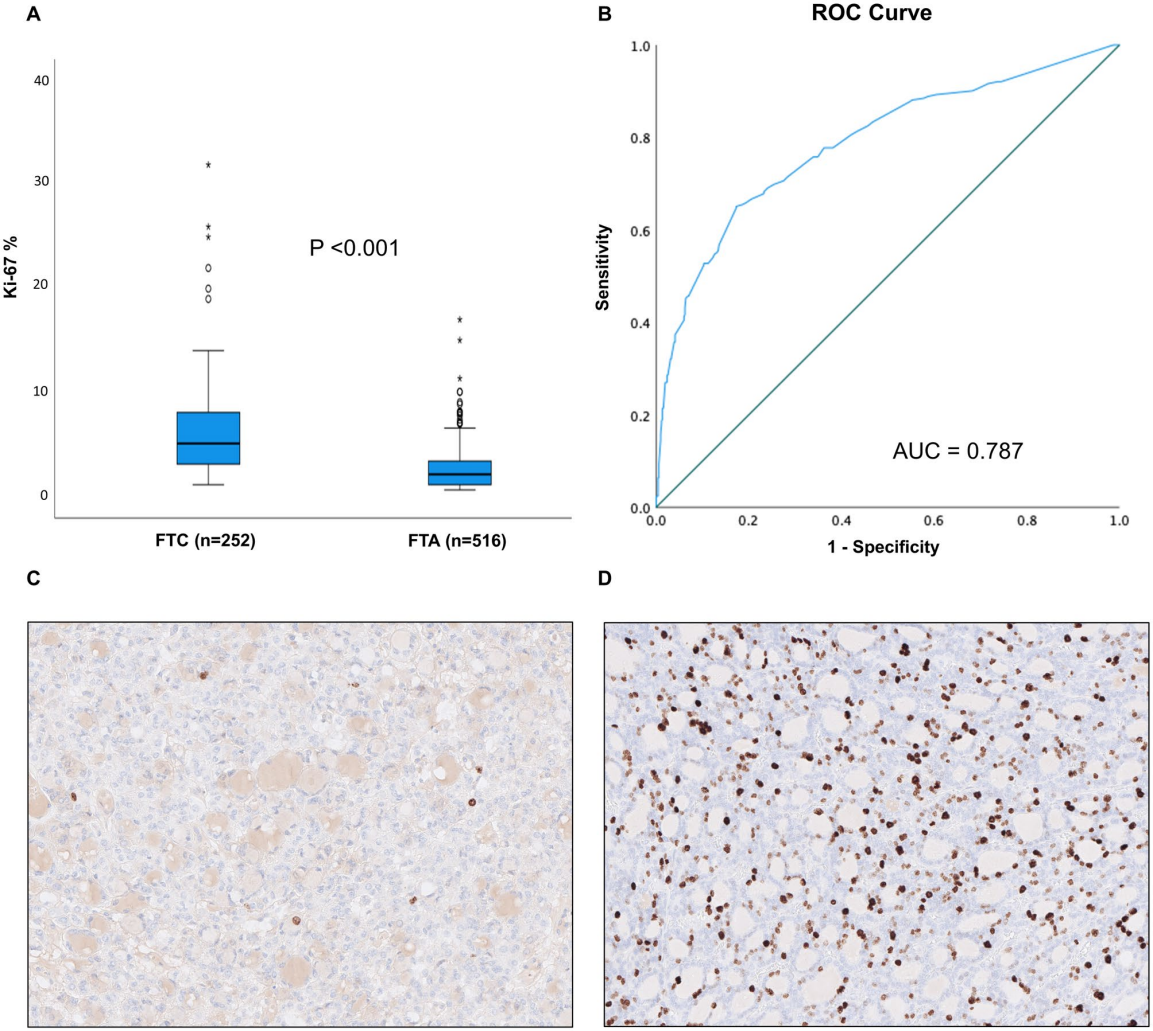
Ki-67 labeling index in follicular thyroid carcinomas (FTC) and follicular thyroid adenomas (FTA). **A** Boxplot presentation of Ki-67 indices in patients with FTC or FTA. Boxes correspond to interquartiles, bars to non-outlier ranges, and bold lines to medians. Mann–Whitney *U*-test showed a significant difference between the groups, *P* < 0.001. **B** The receiver operating characteristic curve (ROC) analysis shows the diagnostic ability of Ki-67 to distinguish patients with FTC from FTA. The analysis showed that a cut-off value of ≥ 4% gave a sensitivity of 65% and a specificity of 83%. The area under curve (AUC) was 0.787. **C** and **D** Illustrative examples of an FTA with a low Ki-67 index (**C**) compared to an FTC with a high Ki-67 index (**D**). The brown nuclei represent positively stained cells and the blue nuclei represent negatively stained cells

The average Ki-67 labeling indices were 2.6% (range 0.5–17) for FTAs, 5.1% (range 1–14) for FT-UMPs, and 5.8% (range 1–32) for FTCs. There were no significant differences in Ki-67 index between different subgroups of FTC (Kruskal–Wallis test, *P* = 0.71) (Table 2). Overall, FTCs displayed higher Ki-67 indices than FTAs (Mann–Whitney *U*, *P* < 0.001) (Fig. 2A) but not higher than FT-UMPs (*P* = 0.30). FT-UMPs exhibited higher Ki-67 indices than FTA (*P* < 0.001). Moreover, a ROC curve analysis revealed a cut-off value of ≥ 4.0% to separate FTC from FTA with a sensitivity and specificity of 65% and 83%, respectively (Fig. 2B). Photomicrographs of follicular thyroid tumors exhibiting high and low Ki-67 labeling indices are presented in Fig. 2C–D.

**Table 2:** Ki-67 index in subgroups of follicular thyroid carcinomas

	**miFTC**	**eaiFTC**	**wiFTC**	Other FTCs *
Number of tumors	120	34	95	3
Mean Ki-67% (range)	5.3 (1–13.5)	5.7 (1–12.5)	6.5 (1–32)	5.7 (5–6.5)

*miFTC* minimally invasive follicular thyroid carcinoma, *wiFTC* widely invasive follicular thyroid carcinoma, *eaiFTC* encapsulated angioinvasive follicular thyroid carcinoma, *FTC* follicular thyroid carcinoma

^*^Two FTCs with a predominant macrofollicular growth pattern and one FTC not otherwise specified

### Prognostic Relevance of the Ki-67 Labeling Index

When assessing the prognostic relevance of the Ki-67 labeling index in FTCs, we first performed a Cox regression for each of the assessed pathological and clinical parameters with either metastasis/recurrence or death of disease as outcome variables. We analyzed factors such as age at surgery, sex, primary tumor size, the Ki-67 labeling index, presence of ETE, capsular invasion, vascular invasion, oncocytic differentiation, and pTNM. All parameters that were significant predictors of metastasis/recurrence and/or death of disease when analyzed separately were analyzed together in a multivariate Cox regression analysis. The results are presented in Table 3 and show that age at surgery, the Ki-67 labeling index, and the presence of ETE are independent predictors of metastatic/recurrent disease (disease-free survival), whereas the Ki-67 labeling index and age at surgery were independent factors foreseeing patients succumbing to disease.

**Table 3:** Univariate and multivariate analyses of clinical and histopathological parameters of the follicular thyroid carcinomas

**Univariate analyses**	**Predictor of metastasis/recurrence**		**Predictor of death of disease**	
	P value	Hazard ratio (CI 95%)	P value	Hazard ratio (CI 95%)
Age at surgery	< 0.001*	1.06 (1.04–1.09)	< 0.001*	1.07 (1.03–1.10)
Tumor size	0.006*	1.02 (1.01–1.03)	0.001*	1.03 (1.01–1.05)
Ki-67	< 0.001*	1.09 (1.05–1.14)	< 0.001*	1.11 (1.05–1.16)
pTNM	< 0.001*	2.62 (1.55–4.42)	0.002*	3.95 (1.64–9.50)
Capsular invasion	0.257	1.72 (0.67–4.36)	0.833	1.14 (0.33–3.94)
Vascular invasion	< 0.001*	3.74 (1.73–8.08)	0.020*	4.33 (1.26–14.87)
Extrathyroidal extension	< 0.001*	8.65 (3.83–19.53)	0.005*	6.33 (1.75–22.84)
Sex, female	0.121	0.62 (0.34–1.14)	0.008*	0.29 (0.12–0.73)
Oncocytic differentiation	0.683	1.15 (0.59–2.24)	0.084	2.24 (0.90–5.60)

**Multivariate analyses**	**Predictor of metastasis/recurrence**		**Predictor of death of disease**	
	P value	Hazard ratio (CI 95%)	P value	Hazard ratio (CI 95%)
Age at surgery	< 0.001*	1.06 (1.03–1.09)	0.005*	1.06 (1.02–1.10)
Tumor size	0.754	1.00 (0.98–1.02)	0.284	1.02 (0.98–1.06)
Ki-67	0.014*	1.08 (1.02–1.15)	0.025*	1.09 (1.01–1.18)
pTNM	0.598	0.79 (0.32–1.92)	0.423	0.56 (0.13–2.34)
Vascular invasion	0.151	1.86 (0.80–4.33)	0.353	1.90 (0.49–7.30)
Extrathyroidal extension	0.001*	5.63 (1.94–16.43)	0.07	4.59 (0.88–23.84)
Sex, female	-	-	0.514	0.68 (0.21–2.17)

Univariate and multivariate analyses were performed to find independent predictors of metastasis/recurrence and independent predictors of death of disease

*CI* confidence interval, *pTNM* Pathological tumor stage according to the American Joint Committee on Cancer (AJCC) Cancer Staging Manual 8^th^ edition

^*^P value < 0.05

**Fig. 3:**
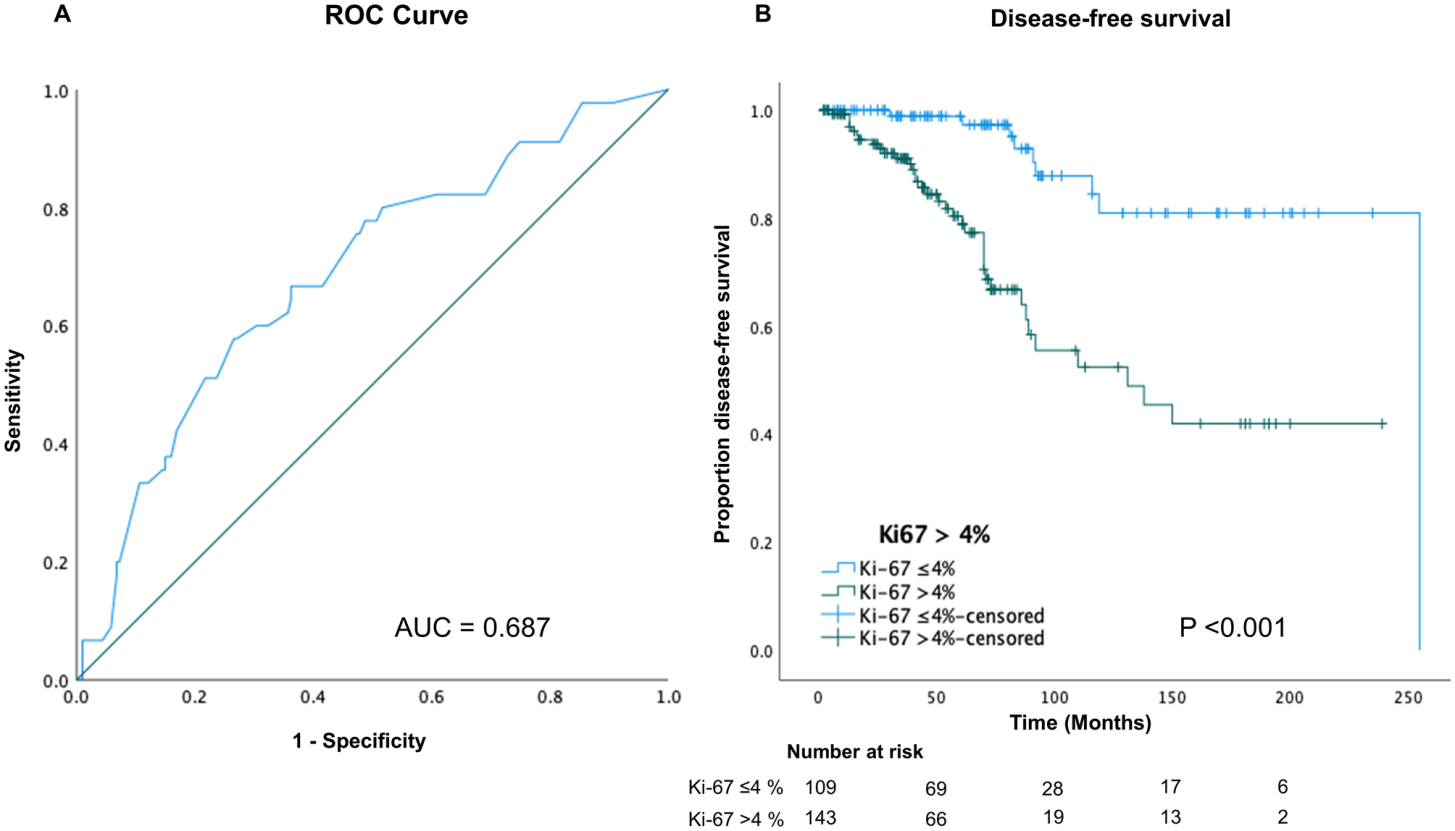
Disease-free survival of FTCs in relation to the Ki-67 labeling index. **A** Receiver operating characteristic (ROC) curve analysis was performed to distinguish which FTCs that have a high risk for metastasis. Selecting a cut-off value of > 4% gave a sensitivity of 80% and a specificity of 48%. The area under curve (AUC) was 0.687. **B** Kaplan–Meier plot illustrating the difference in time to metastatic/recurrent disease between tumors with Ki-67 index > 4% compared to tumors with Ki-67 ≤ 4% (log-rank test, < 0.001). The number at risk for each time point is included below the graph

A ROC curve analysis was performed to establish an optimal cut-off for the detection of FTCs that would subsequently behave metastatic/recurrent and generated a Ki-67 labeling index of > 4%, with a sensitivity of 80% and a specificity of 48% (Fig. 3A). To build on this, a survival analysis with the endpoint time to metastatic event/recurrence triaged FTCs with a Ki-67 cut-off of 4% into two separate risk groups (log-rank test, *P* < 0.001) (Fig. 3B), and a similar outcome was noted when the endpoint was death of disease (log-rank, *P* = 0.005).

### TNM Stratification

**Fig. 4:**
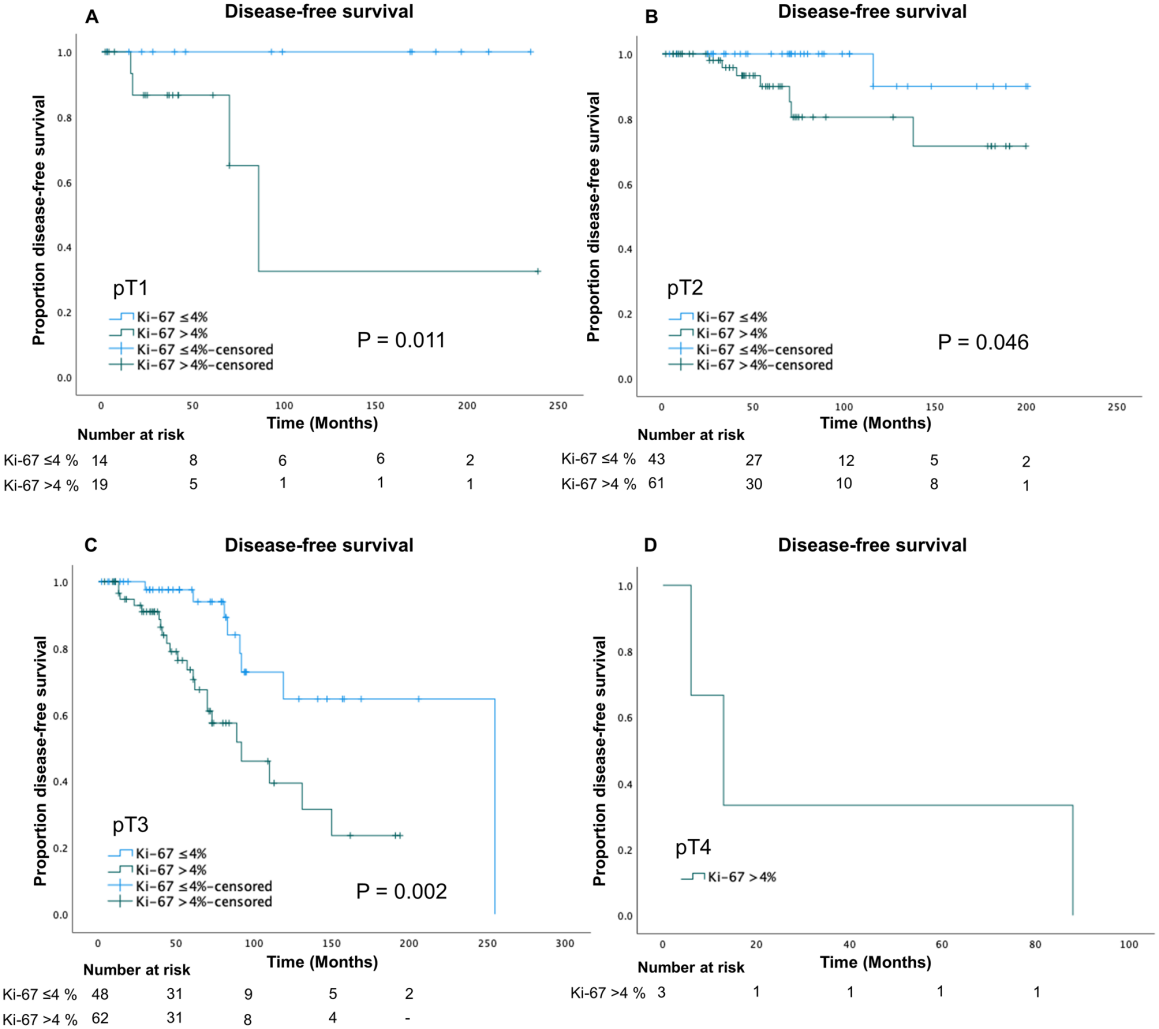
Disease-free survival within T stage groups in relation to Ki-67 labeling index. Patients with Ki-67 indices > 4% were compared to patients with Ki-67 indices ≤ 4% regarding time to metastatic/recurrent disease. Significant differences were found for pT1 (**A**), pT2 (**B**), and pT3 (**C**) tumors (log-rank, *P* = 0.011, *P* = 0.046, and *P* = 0.002, respectively). The analysis was not performed on pT4 (**D**) tumors since all lesions had a Ki-67 index > 4%. The number at risk for each time point is included below each graph

The TNM system is a globally accepted classification for prognosticating human cancers, and all auxiliary analyses aimed to improve the risk assessment of tumors should therefore be compared to the current gold standard. The pT stage takes the primary tumor size and the extent of the tumor into consideration, making this parameter quite straightforward to establish and reproduce between institutions. We therefore analyzed the outcome of the Ki-67 labeling index within each pT stage group (pT1-4) for all FTCs (Table 4). In terms of clinical outcome, the rate of metastases/recurrence within each pT stage group was significantly higher in pT3-4 FTCs compared with pT1-2 FTCs (chi-square *P* < 0.001), which was also true for the outcome “death of disease” (chi-square *P* = 0.009). The Ki-67 labeling index was not significantly higher in pT3-4 FTCs than in pT1-2 tumors (Mann–Whitney *U*, *P* = 0.64). Kaplan–Meier analyses for FTCs within individual pT staging groups revealed a shorter time to metastatic events/recurrence for FTCs with a Ki-67 labeling index of > 4% as compared to ≤ 4% for stages pT1, pT2, and pT3, while all pT4 tumors exhibited a Ki-67 > 4% (Fig. 4).

**Table 4:** Clinical and histological data for FTC tumors grouped by T stage

	**pT1**	**pT2**	**pT3**	**pT4**
Number of tumors	33	104	110	3
Female:male	28:5	80:24	67:43	1:2
Oncocytic differentiation, *n*	13 (39.4%)	33 (31.7%)	25 (22.7%)	3 (100%)
Metastasis/recurrent disease, *n*	4 (12.1%)	8 (7.7%)	30 (27.3%)	3 (100%)
Death of disease, *n*	0 (0%)	5 (4.8%)	12 (10.9%)	2 (66.7%)
miFTC, *n*	24	57	38	0
wiFTC, *n*	2	32	57	3
eaiFTC, *n*	6	13	15	0
Other FTC, *n*	1	2	0	0
Mean Ki-67, % (range)	5.3 (1–13.3)	5.6 (1–20)	6 (1–32)	12.5 (5–22)
Mean age at surgery, (range)	49.9 (17–80)	51.7 (13–91)	59.7 (10–92)	75.7 (70–83)
Mean tumor size, mm (range)	16.2 (9–20)	31.2 (21–40)	57.7 (18–120)	81 (53–100)

*FTC* follicular thyroid carcinoma, *miFTC* minimally invasive follicular thyroid carcinoma, *wiFTC* widely invasive follicular thyroid carcinoma, *eaiFTC* encapsulated angioinvasive follicular thyroid carcinoma, *pT1-4* pathological tumor stage according to American Joint Committee on Cancer (AJCC) Cancer Staging Manual 8^th^ edition, *n* sample size

### Associations to TERT Promoter Mutation and Gene Expression

When comparing *TERT* promoter mutation status or the presence of *TERT* gene expression with Ki-67 indices > 4% in FTCs using cross-tabulation, significant correlations to *TERT* promoter mutations (*p* = 0.04, Pearson chi-square) and the presence of *TERT* gene expression (< 0.001, Pearson chi-square) were noted. Further analyses of quantitative *TERT* gene expression levels revealed a significant correlation between a Ki-67 index of > 4% and absolute *TERT* expression levels (*p* < 0.001, Mann Whitney *U*). No correlation was found when comparing *TERT* promoter mutational status with the absolute numbers of Ki-67 proliferation indices (*p* = 0.15, Mann Whitney *U*).

### Reproducibility of the Ki-67 Labeling Index over Time

At our department, the Ki-67 stain was originally performed manually before changed to automatic staining, and the study period also included different antibody clones. One pathologist (CCJ) therefore reviewed and recounted 30 cases from different time periods (including manually and automatically stained slides, as well as slides stained with the Mib-1 and CONFIRM clones) to check whether the Ki-67 results were concordant today as when they were originally reported. The mean deviation between recounting results and original scores was 0.12 percentage points, ranging from − 1 to + 1 percentage points. Setting the cut-off at 4%, only one case (3%) changed groups (an FTA with a Ki-67 index of 4% in the original report, and 4.2% in the recounting). A Cohen’s Kappa score of 0.918 was obtained when comparing recounting results with the original pathology reports, indicating adequate agreement irrespectively of the time period.

## Discussion

The arrival of next-generation sequencing techniques has the potential to improve the diagnostic and prognostic capacity of modern pathology laboratories, potentially allowing the diagnostician to fine-tune the clinical work-up of follicular thyroid tumors. However, as many of these analyses are still expensive and labor-intense and demand stringent validation, histology in combination with standardized and well-established immunohistochemical markers is still the cornerstone in endocrine pathology. Ki-67 is a nuclear antigen that is intimately associated with the tumoral proliferative capacity, and most endocrine pathologists in tertiary centers are well familiar with the interpretation and scoring of this marker. As subsets of follicular thyroid tumors may be notoriously difficult to diagnose and prognosticate even in the hand of experienced thyroid cancer experts, we sought to investigate the value of the Ki-67 labeling index in this aspect by a retrospective analysis of the hitherto largest tumor cohort to date with available long-term follow-up data.

In our series, we found that the Ki-67 labeling index was higher overall in FTCs than in FTAs, and a cut-off at 4% showed a 65% sensitivity and an 83% specificity towards identifying FTC and excluding FTA. Since FTAs are more commonly encountered than FTCs in clinical practice, any marker with the ambition to separate these entities need to exhibit high specificity to avoid an excessive number of false positives. In this aspect, a cut-off at 4% was optimal according to the ROC analysis. However, the diagnostic process is based on gold standard histopathological examination, and the observed overlap in Ki-67 indices between FTCs and FTAs makes this marker of limited value in the postoperative setting. Even so, our data are in line with a previous FNAB study in which a Ki-67 cut-off value of 5% was suggested to identify FTCs, thereby somewhat reinforcing the idea that this marker could exhibit diagnostic properties on the preoperative level [24].

In terms of prognostication, Ki-67 was an independent predictor of metastatic events and death of disease in our cohort. Moreover, FTCs with a Ki-67 labeling index > 4% were significantly associated with metastatic/recurrent disease and poorer patient outcome. This was also true for FTCs stratified within the individual pT staging group, suggesting that Ki-67 holds prognostic value independent of primary tumor size. This could be particularly beneficial when assessing smaller FTCs (pT1a/b), as these patients are considered low risk and might therefore not receive additional surgery or ablation radioiodine treatment [30]. In future studies, it would be interesting to see if patients with pT1a/b FTCs and a Ki-67 index of > 4% could benefit from adjuvant treatment, given the increased risk of poor outcome indicated from our data. Moreover, a comparison to molecular markers that may indicate a worse prognosis in FTCs should also be performed to investigate if the Ki-67 index in combination with molecular analyses may be a way forward to individualize the prognostication of these lesions [26, 31–33]. Indeed, in this study, FTCs with Ki-67 indices > 4% were significantly associated with *TERT* promoter mutations as well as with the presence of *TERT* gene expression. The notion that FTCs with a Ki-67 index > 4% are overrepresented in terms of *TERT* aberrancies thus supports our conclusion that these lesions may be associated with a poorer prognosis. Indeed, previous studies indicate that both *TERT* promoter mutations and *TERT* mRNA expression indicate worse clinical outcome in differentiated thyroid carcinoma and may also be associated to higher Ki-67 indices [29, 34–36].

The study is not without limitations. Given the retrospective nature and the initial lack of standardization in terms of which FTAs were analyzed, there is a risk of selection bias when the responsible pathologist more likely ordered a Ki-67 immunohistochemical stain for equivocal cases or large FTAs than for straightforward tumors. Even so, the large sample size, the long follow-up time, the uniform scoring procedure (counting 2000 cells in hot spot areas), and the reproducibility over time make the results valid from a clinical screening perspective. Moreover, even though we re-analyzed subsets of cases stained during different time periods to exclude tissue handling bias, we cannot fully exclude that differences in the laboratory protocols could affect the staining quality and hence the ensuing interpretation. Furthermore, apart from our analyses of *TERT* promoter mutations and expression in this study, we lack comprehensive molecular data for the cohort, making comparisons to current genomic classifiers (such as ThyroSeq or Afirma) unfeasible [37, 38]. As modern pathology laboratories will probably see a future integration of histology, immunohistochemistry, and molecular genetics in order to provide the most detailed prognostication in terms of FTCs, a combination of sequencing data and mRNA/protein expression–based analyses will probably be needed to obtain superior sensitivity and specificity in clinical routine practice. For example, the addition of NRAS Q61R mutation–specific immunohistochemistry could have added value to our work, and future efforts will be needed to correlate *RAS* mutational status to the proliferative abilities of follicular thyroid tumors [39].

There might also be several FTCs in this cohort classified by the 2004 or 2017 WHO criteria that could be considered as differentiated high-grade thyroid carcinomas (DHGTCs), a group of thyroid malignant tumors characterized by a well-differentiated growth pattern, but with an elevated mitotic count and/or tumor necrosis [40]. Indeed, Hiltzik and colleagues recently proposed the entity “non-solid/trabecular/insular type of poorly differentiated carcinoma” which had high-grade features (increased mitoses and tumor necrosis) [41], and a Ki-67 index of ≥ 4% in tumors fulfilling the “Hiltzik criteria” was an independent predictor of cause-specific survival in an independent series [42]. When manually reading pathology reports from cases included in the current study, we found no FTC cases that presented with extensive elevations of the mitotic count and/or tumor necrosis. However, this could be due to reporting bias, since the DHGTC criteria were not established at the time of diagnosis. Even so, it makes sense that highly proliferative follicular thyroid carcinomas associated with *TERT* gene aberrancies are indeed high-grade lesions, which is also the case in this study. Therefore, a thorough histological overhaul of our cohort using the upcoming 2022 WHO criteria for DHGTCs would constitute an interesting follow-up project [40].

Finally, although our data indicate a promising role for Ki-67 in the risk assessment of follicular thyroid tumors, a general recommendation to include the Ki-67 labeling index in the histopathological work-up of follicular thyroid tumors necessitates standardization of interpretation and international calibrations, and therefore, multi-center studies are required—not least since different institutions have different tissue handling and immunohistochemical protocols that may affect the interpretation of the staining.

We conclude that the Ki-67 labeling index is a potentially valuable marker for the prognostication of FTCs. If sufficiently reproduced in international series, the implementation in clinical routine histopathological assessments of follicular thyroid tumors should be considered.

## Data Availability

The datasets generated during the current study will be available upon reasonable request.
